# A Case Series on Rhabdomyolysis in a Tertiary Care Centre

**DOI:** 10.7759/cureus.90989

**Published:** 2025-08-25

**Authors:** Naveenkumar Viswanathan, Sandhiya T, Sahasyaa Adalarasan, Yogesh S

**Affiliations:** 1 General Surgery, Cwm Taf Morgannwg University Health Board, Merthyr Tydfil, GBR; 2 Medicine and Surgery, Madras Medical College, Chennai, IND; 3 Internal Medicine, Madras Medical College, Rajiv Gandhi Government General Hospital, Chennai, IND

**Keywords:** acute kidney injury, muscle injury, non-traumatic rhabdomyolysis, rhabdomyolysis, traumatic rhabdomyolysis

## Abstract

Rhabdomyolysis is a condition characterized by the breakdown and necrosis of muscle tissue, leading to the release of intracellular components into the bloodstream. It results from the destruction of the sarcolemmal membrane, which allows these substances to enter circulation. The clinical presentation can vary widely - from asymptomatic elevations in serum muscle enzymes to severe complications, such as volume depletion, metabolic and electrolyte imbalances, and acute kidney injury (AKI). In this report, we present three cases of rhabdomyolysis treated at Rajiv Gandhi Government General Hospital, Chennai, India, each exhibiting a distinct range of clinical manifestations.

## Introduction

Rhabdomyolysis refers to the disintegration of striated muscle, resulting in the release of intracellular contents into the bloodstream. It is most commonly caused by skeletal muscle injury, which may be either traumatic or non-traumatic [1]. Skeletal muscle comprises approximately 40% of total body mass and can store substantial intracellular materials, which may overwhelm the body’s clearance mechanisms when released suddenly.

Acute rhabdomyolysis may present with muscle soreness and reddish-brown urine. Diagnostic indicators of rhabdomyolysis-related acute kidney injury (AKI) include pigmented granular casts in the urine, a positive blood reading on dipstick testing, and the absence of red blood cells on urine microscopy. However, definitive diagnosis requires elevated serum creatine kinase (CK) levels and the presence of myoglobin in the urine [2,3].

The correlation between CK levels and the risk of AKI is limited. While CK levels below 20,000 U/L are generally associated with a lower risk of AKI, levels as low as 5,000 U/L may still lead to kidney injury when accompanied by other factors, such as sepsis or intravascular volume depletion [4].

Management of rhabdomyolysis centers on the prevention of AKI and the correction of electrolyte imbalances. This is typically achieved through aggressive intravenous fluid therapy to promote renal perfusion and myoglobin clearance. In more severe cases, interventions such as urine alkalinization, electrolyte correction, and dialysis may be required [5]. Early recognition and appropriate treatment are essential to minimize complications such as renal failure, cardiac arrhythmias, and mortality.

## Case presentation

Case 1

A 33-year-old male presented with a history of an unknown bite on the right foot sustained at Kovalam Beach, Chennai, India. He developed generalized myalgia and was initially taken to a private hospital, where primary care was administered before being referred to Rajiv Gandhi Government General Hospital (RGGGH).

On admission to RGGGH, the patient was conscious, oriented, and afebrile. There were no signs of pallor, icterus, cyanosis, clubbing, pedal edema, or lymphadenopathy. Vital signs were stable, and systemic examination was unremarkable. Local examination of the bite site revealed fang marks without signs of cellulitis. His initial blood parameters are listed in Table 1.

**Table 1:** Laboratory values of investigations in Case 1 SGOT: Serum Glutamic Oxaloacetic Transaminase, SGPT: Serum Glutamic Pyruvic Transaminase, CK: Creatine Kinase, CK-MB: Creatine Kinase-Muscle/Brain, ABG: Arterial Blood Gas

Investigations	Results	Reference Range
Total count	16 x 10^3^ IU/L	4-11 x 10^3^ IU/L
Hemoglobin	14.1 g/dL	13.5-17.5 g/dL
Urea	38 mg/dL	7-21 mg/dL
Creatinine	0.9 mg/dL	0.7-1.3 mg/dL
SGOT	284 IU/L	5-40 U/L
SGPT	234 IU/L	7-56 U/L
Sodium	135 mEq/L	135-145 mEq/L
Potassium	5.7 mEq/L	3.5-5 mEq/L
CK	16,500 IU/L	39-308 U/L
CK-MB	606 IU/L	0-25 IU/L
Urine Myoglobin	Positive	Negative
pH	7.4	7.35-7.45

The patient was managed with intravenous fluids, ample oral hydration, and started on forced alkaline diuresis. Serial monitoring of vital signs and urine output was performed. His laboratory parameters gradually improved. A total of nine cycles of forced alkaline diuresis were administered. His CK and CK-MB levels were reduced to 78 IU/L and 50 IU/L, respectively. Forced alkaline diuresis was discontinued, and the patient was discharged in stable condition.

Case 2

A 24-year-old female, a known case of seizure disorder, presented with a generalized tonic-clonic seizure (GTCS) lasting 15 minutes without regaining consciousness. She was intubated and managed according to the status epilepticus protocol, including the administration of antiepileptics and benzodiazepines. Initial blood parameters were within normal limits.

She remained under continuous sedation due to intermittent seizure episodes. On the following day, her renal parameters began to deteriorate, and her urine output decreased. Laboratory values on day two are detailed in Table 2.

**Table 2:** Laboratory values of investigations done in Case 2 CK: Creatine Kinase, CK-MB: Creatine Kinase-Muscle/Brain, ABG: Arterial Blood Gas

Investigations	Results	Reference Range
Total count	11 x 10^3^ IU/L	4-11 x 10^3^ IU/L
Hemoglobin	13.1 gm/dL	13.5-17.5 g/dL
Urea	74 mg/dL	7-21 mg/dL
Creatinine	2.3 mg/dL	0.7-1.3 mg/dL
Sodium	135 mEq/L, 5.8 mEq/L	135-145 mEq/L
Potassium	5.8 mEq/L	3.5-5 mEq/L
CK	5400 IU/L	39-308 U/L
CK-MB	489 IU/L	0-25 IU/L
Urine Myoglobin	Positive	Negative
pH	7.28	7.35-7.45

Despite adequate hydration and correction of hyperkalemia, her renal function continued to decline, and she developed oliguria. Hemodialysis was initiated, and a total of four cycles were administered. Subsequently, her renal parameters and urine output improved. The patient showed symptomatic improvement, was extubated, and was monitored for further seizure activity. She was discharged with a prescription for antiepileptic medications.

Case 3

A 32-year-old male presented to RGGGH with severe myalgia and reduced urine output after returning from a one-week trip to North India during the month of May. He traveled back to Chennai by train, with a journey time of approximately 36 hours. He reported experiencing a headache and intense muscle pain during the return journey and sought immediate care at RGGGH upon arrival.

On examination, the patient’s vital signs were stable, though he was febrile (temperature: 103°F) and dehydrated. He was also oliguric. His laboratory findings are shown in Table 3.

**Table 3:** Laboratory values of investigations done in Case 3 SGOT: Serum Glutamic Oxaloacetic Transaminase, SGPT: Serum Glutamic Pyruvic Transaminase, CK: Creatine Kinase, CK-MB: Creatine Kinase-Muscle/Brain, ABG: Arterial Blood Gas

Investigations	Results	Reference Range
Total count	14 x 10^3^/UL	4-11 x 10^3^ IU/L
Hemoglobin	16.1 gm/dL	13.5-17.5 g/dL
Urea	94 mg/dL	7-21 mg/dL
SGOT	284 IU/L	5-40 IU/L
SGPT	234 IU/L	7-56 IU/L
Creatinine	3.1 mg/dL	0.7-1.3 mg/dL
Sodium	135 mEq/L	135-145 mEq/L
Potassium	5.7 mEq/L	3.5-5 mEq/L
CK	8000 IU/L	39-308 U/L
CK-MB	506 IU/L	0-25 IU/L
Urine Myoglobin	Positive	Negative
pH	7.3	7.35-7.45

A diagnosis of heat stroke with multiorgan dysfunction syndrome and rhabdomyolysis was made. He was promptly treated with intravenous fluids and cooling blankets. Due to acute renal failure, he was initiated on hemodialysis. After five cycles of hemodialysis, his renal parameters and urine output significantly improved. Other abnormal laboratory values also normalized, and the patient was discharged in stable condition.

## Discussion

Rhabdomyolysis may result from traumatic or non-traumatic causes, each leading to muscle injury via different mechanisms. Traumatic triggers include crush injuries, prolonged immobilization, and intense or unaccustomed physical exertion. Non-traumatic causes are more varied and include medications (e.g., statins, antipsychotics, zidovudine, lithium), substance abuse (cocaine, alcohol, ecstasy), seizures, heat stroke, viral infections (e.g., influenza, HIV), toxins (venoms, alcohol), metabolic disorders (hypothyroidism, diabetes, electrolyte imbalances), inflammatory myopathies (dermatomyositis, polymyositis), and genetic conditions such as McArdle disease and malignant hyperthermia [6,7].

Pathophysiologically, muscle injury leads to elevated intracellular calcium, activating enzymes that break down muscle fibers and release myoglobin into circulation. Myoglobin can cause direct renal tubular damage, while inflammation, fluid shifts, and vasoconstriction further compromise renal function, increasing the risk of acute kidney injury. Systemic effects such as hyperkalemia, metabolic acidosis, and disseminated intravascular coagulation (DIC) can also occur [8].

This case series illustrates three distinct rhabdomyolysis presentations: envenomation at the beach, seizures, and heat stroke. All cases featured elevated CK and myoglobinuria, with varying degrees of renal impairment. Aggressive management - including fluid resuscitation, forced alkaline diuresis, and hemodialysis - proved effective in reversing renal damage and achieving recovery.

These findings highlight the need for early recognition and intervention in suspected rhabdomyolysis, especially in patients presenting with myalgia, dark urine, or oliguria in the presence of risk factors. Prompt treatment significantly reduces the risk of complications and improves clinical outcomes [9,10].

## Conclusions

The prognosis of rhabdomyolysis largely depends on the underlying cause, the degree of muscle injury, and the timeliness of treatment. Understanding the pathophysiology and potential complications associated with rhabdomyolysis is essential for healthcare providers to ensure optimal patient outcomes and minimize the risk of long-term morbidity.

This case series highlights the importance of recognizing and managing rhabdomyolysis promptly to prevent AKI and other complications. Effective interventions, including hydration and hemodialysis, can significantly improve patient outcomes.
